# Association of Sleep Patterns with Type 2 Diabetes Mellitus: A Cross-Sectional Study Based on Latent Class Analysis

**DOI:** 10.3390/ijerph20010393

**Published:** 2022-12-26

**Authors:** Mengdie Liu, Wali Lukman Ahmed, Lang Zhuo, Hui Yuan, Shuo Wang, Fang Zhou

**Affiliations:** 1 School of Nursing, Xuzhou Medical University, Xuzhou 221004, China; 2 School of Public Health, Xuzhou Medical University, Xuzhou 221004, China

**Keywords:** diabetes mellitus, sleep, latent class analysis

## Abstract

Sleep duration, sleep quality and circadian rhythm disruption indicated by sleep chronotype are associated with type 2 diabetes. Sleep involves multiple dimensions that are closely interrelated. However, the sleep patterns of the population, and whether these sleep patterns are significantly associated with type 2 diabetes, are unknown when considering more sleep dimensions. Our objective was to explore the latent classes of sleep patterns in the population and identify sleep patterns associated with type 2 diabetes. Latent class analysis was used to explore the best latent classes of sleep patterns based on eleven sleep dimensions of the study population. Logistic regression was used to identify sleep patterns associated with type 2 diabetes. A total of 1200 participants were included in the study. There were three classes of sleep patterns in the study population: “circadian disruption with daytime dysfunction” (class 1), “poor sleep status with daytime sleepiness” (class 2), and “favorable sleep status” (class 3). After controlling for all confounding factors, people in class 2 have significantly higher prevalence of type 2 diabetes than those in class 3 (OR: 2.24, 95% CI 1.26–4.00). Sleep problems have aggregated characteristics. People with sleep patterns involving more or worse sleep problems have higher significantly prevalence of T2DM.

## 1. Introduction

Diabetes mellitus is a chronic metabolic disease characterized by hyperglycemia. There were 537 million adults aged 20–79 years with diabetes worldwide in 2021, of which type 2 diabetes mellitus (T2DM) accounted for more than 90% [1]. As the disease progresses, it impairs the individual’s physical (heart, kidneys, blood vessels and nerves, etc.), psychological and social functions, reduces quality of life and increases disability and mortality [2,3,4]. Although researchers have been trying to find ways to cure T2DM, to date, it remains an incurable, chronic, lifelong disease [5]. Therefore, the factors associated with T2DM are of great concern, especially modifiable behavioral and lifestyle factors.

Multiple behavioral and lifestyle factors, such as sleep, physical activity and eating behavior, have been confirmed to be associated with T2DM [6,7,8,9], and T2DM is increasingly seen in children, adolescents and younger adults due to the prevalence of unhealthy behaviors and lifestyles [10]. In addition, it has been found that behavioral lifestyle interventions such as weight loss, diet and physical activity reduce the risk of diabetes by approximately 50%; furthermore, this risk reduction is sustained for many years after interventions [11]. Therefore, the accurate identification of modifiable behavioral and lifestyle factors associated with T2DM is pivotal in management of the disease.

Sleep is a vital physiological process involved in regulating many physiological functions of the human body [12]. Most existing studies have focused on the relationship between one particular aspect of sleep and T2DM, and the results showed that excessive or insufficient sleep duration, poor sleep quality or circadian rhythm disruption indicated by sleep chronotype and social jet lag (SJL) were associated with T2DM [13,14,15,16]. However, sleep health follows a multidimensional pattern of sleep-wakefulness, and the characteristics of good sleep health involve multiple aspects of sleep, such as subjective satisfaction, appropriate timing, adequate duration, high efficiency, and sustained alertness during waking hours [17]. In addition, there may be complex internal associations between various aspects of sleep, such as sleep duration, which may be related to sleep chronotype, and sleep quality at night may affect daytime sleep and function. A few studies have noted these issues and further analyzed the possible association between different sleep patterns and T2DM. For example, Zhai et al. found that compared to those with an intermediate chronotype and 7–8 h of night sleep duration, individuals with a night chronotype and >9 h of night sleep duration had the highest risk of T2DM [18]. A meta-analysis showed that short objective sleep duration (<6 h) was significantly associated with T2DM only in the presence of insomnia symptoms [19]. However, the formation of sleep patterns in these studies is predefined and does not adequately reflect sleep patterns in the real world.

Latent class analysis (LCA) is a statistical method used to identify populations with similar characteristics based on the selection propensity of individuals in different manifest variables. An advantage of this approach is that the grouping originates from data; therefore, the categories are not predefined and are not limited by current conceptual frameworks [20]. The classification of sleep patterns using LCA can comprehensively analyze various sleep dimensions and their complex real-world interrelations. A previous study used the LCA to classify the population into three classes based on sleep dimensions and further analyzed the relationship between each class and diabetes [21]. However, the sleep dimensions in the study only involved sleep quality, sleep duration, sleep latency, sleep disturbances, daytime sleepiness and daytime dysfunction, and the study did not differentiate between type 1, type 2 and other types of diabetes. In fact, there are fundamental differences between the different types of diabetes that need to be analyzed separately. Hence, further research is needed to investigate the following questions: (1) based on comprehensive sleep dimensions, what sleep patterns exist in real-world populations? and (2) which sleep pattern is associated with T2DM?

We hypothesized that through a comprehensive analysis of people’s sleep dimensions, accurate classification of their sleep patterns could be made, and the prevalence of T2DM is closely associated with sleep patterns involving more or worse sleep problems and poor sleep behaviors. This study aims to (1) comprehensively consider various aspects of sleep, including subjective sleep quality, sleep latency, sleep duration, sleep efficiency, sleep disturbances, daytime dysfunction, daytime sleepiness, bedtime procrastination, sleep chronotype, SJL, and use of sleep medication, and explore the latent classes of sleep patterns based on LCA; and (2) identify the sleep patterns associated with T2DM.

## 2. Materials and Methods

### 2.1. Study Design and Participants

This was a cross-sectional study conducted between March and July 2022 in Xuzhou, Jiangsu Province, China. The participants were recruited while receiving a conventional physical at the community hospital, and a face-to-face questionnaire survey was administered. The inclusion criteria were as follows: aged 18 years or older and volunteered to participate in the study. Meanwhile, participants who were diagnosed with type 1 diabetes, gestational diabetes, or other specific types of diabetes, who were in a state of secondary hyperglycemia due to diseases (except diabetes) or medications, or who were unable to cooperate in completing the questionnaire, were excluded from the study.

Participants were selected using a multistage random sampling method. Stage 1: first, all administrative districts of Xuzhou City were numbered, and three administrative districts were randomly selected from them by drawing lots. Stage 2: two communities were randomly selected by drawing lots from the communities under the jurisdiction of each district, yielding a total of six communities as the basic sampling unit. Stage 3: a convenience sampling method was used to recruit more than 200 participants from each community who met the inclusion criteria.

### 2.2. Assessment of Sleep

The Chinese version of the Pittsburgh Sleep Quality Index (PSQI) was used to measure seven domains of sleep, namely, subjective sleep quality, sleep latency, sleep duration, sleep efficiency, sleep disturbances, use of sleep medication and daytime dysfunction, with a Cronbach’s alpha coefficient of 0.84 [22]. There are 19 items in the PSQI, and the scores in each of the seven domains are weighted equally from 0 to 3, with higher scores indicating more severe complaints. According to a previous study, the scores of each dimension were divided into 2 categories: good (score ≤ 1) and poor (score ≥ 2) [23].

The Chinese version of the Epworth sleepiness scale (ESS) was used to measure daytime sleepiness, with a Cronbach’s alpha coefficient of 0.81 [24]. The questionnaire consists of 8 items, each with a score of 0 to 3, for a total score of 0 to 24. The higher the score, the more pronounced the tendency to sleepiness. A score of ≥10 was defined as excessive daytime sleepiness [25].

The Chinese version of the Bedtime Procrastination Scale (BPS) was used to measure the propensity of individuals to delay bedtime when they can freely choose, that is, bedtime procrastination, and the Cronbach’s alpha coefficient was 0.91 [26]. The scale consists of 9 items; each item is scored on a 5-point Likert scale, and the total score ranges from 9 to 45. Higher scores indicated more severe bedtime procrastination. According to a previous study, we divided participants into low-moderate and high bedtime procrastinators based on the cutoff point of the 75th percentile of BPS score in the study population [27].

The Chinese version of the Munich Chronotype Questionnaire (MCTQ), performed in collaboration with the original authors of the MCTQ, measures sleep chronotype and SJL [28]. The internal circadian rhythm of an individual is regulated by the supraoptic nucleus in the brain, which is known as the “biological clock”. Due to the complex influence of the internal and external environment, individuals exhibit specific subjective preferences for their sleep-wake time, which is the sleep chronotype [29]. SJL arises precisely because of the mismatch between biological and social clocks [30]. The questionnaire separately assessed the habitual sleep of individuals on workdays and free days and calculated the respective sleep midpoints, which are intermediate points between the start and end of the sleep period. The midpoint of sleep on free days minus the midpoint of sleep on workdays is used to determine the SJL, and the absolute value was used in our study. Referring to a previous study, we used 1 h as the cutoff to indicate whether the SJL existed [31]. The sleep chronotype was assessed by correcting for the midpoint of sleep on free days (MSFsc), and details of the calculation can be found at www.thewep.org (accessed on 5 October 2022). According to a previous study, participants were divided into morning and night sleep chronotypes using the 75th percentile of MSFsc as the cutoff point [32].

### 2.3. Assessment of T2DM

Participants who had a fasting blood glucose ≥ 7.0 mmol/L, or 2 h postprandial blood glucose ≥ 11.1 mmol/L, or HbA1c ≥ 6.5%, or self-reported previous diagnosis of diabetes by a healthcare professional were defined as having diabetes [33]. Patients with types of diabetes other than T2DM were excluded.

### 2.4. Possible Covariates

Self-designed questions and questionnaires with good reliability and validity were used to collect information, namely, sociodemographic characteristics (age, sex, height, weight, waist circumference, marital status, education, and annual household income), behavioral lifestyle (smoking, alcohol consumption, physical activity level, and eating behavior), personal medical history (hypertension, dyslipidemia, coronary heart disease, stroke, arthritis, osteoporosis, thyroid disease, and cancer), mental psychological state (anxiety, depression, stress, and neuroticism), and family history of diabetes. Because all the respondents had established electronic medical records at community hospitals, information on personal medical history and diabetes was obtained from the community hospital database.

The Chinese version of the International Physical Activity Questionnaire—Short Form (IPAQ-SF) has good reliability and validity for measuring physical activity levels [34]. The scale was developed by the International Physical Activity Measurements Group to report four specific activity types in the last 7 days, including vigorous-intensity activities, moderate-intensity activities, walking, and sitting. Participants were classified into low, moderate, and high levels of physical activity based on the guideline criteria [35].

The Chinese version of the Dutch Eating Behavior Questionnaire (DEBQ) is a 33-item questionnaire measuring eating behavior, with each item rated on a 5-point Likert scale [36]. The questionnaire consists of three subscales: restrained eating, emotional eating, and external eating, each containing 10, 13, and 10 items, respectively. The Cronbach’s alpha coefficients were 0.89, 0.96, 0.87, and 0.91 for restrained eating, emotional eating, external eating, and the total scale, respectively. Restrained eating refers to restricted conscious behavior during eating. Emotional eating is the tendency to use food to cope with psychological problems or to alleviate distress. External eating refers to the frequency of eating in response to external stimuli, such as the appearance and smell of food. Higher scores indicated a higher propensity for each behavior.

The Chinese version of the Depression, Anxiety and Stress Scale-21 (DASS-21) consists of 21 items with three subscales consisting of 7 items in each subscale to measure depression, anxiety, and stress separately [37]. The Cronbach’s alpha coefficients were 0.83, 0.80, 0.82, and 0.92 for depression, anxiety, stress, and the total scale, respectively. Each item was scored with equal weight from 0 to 3. Scores for depression, anxiety, and stress were calculated by summing the scores for the relevant items, with higher scores indicating higher severity for each dimension.

The neuroticism subscale from the brief version of the Chinese Big Five Personality Inventory (CBF-PI-B) developed by Wang et al. was used to measure the level of neuroticism [38]. The Cronbach’s alpha coefficient of the neuroticism subscale was 0.81. The scale consists of 8 items, each scored using a 6-point Likert scale. Higher scores indicated higher levels of neuroticism.

### 2.5. Statistical Analysis

SPSS (version 26.0, IBM, Armonk, NY, USA) was used for all statistical tests except the LCA. Continuous and categorical variables were expressed as the means ± standard deviations (SDs) and numbers (percentages), respectively. The *t* tests, chi-square tests, or nonparametric tests were used for comparisons between groups, followed by post hoc tests.

Mplus (version 8.0, MUTHEN & MUTHEN, Los Angeles, CA, USA) was used to conduct LCA. We examined the model fit indices: Akaike Information Criteria (AIC), Bayesian Information Criteria (BIC), sample-size-adjusted BIC (aBIC), entropy, mean class assignment probabilities, the Vuong-Lo-Mendell-Rubin (LMR) test, and the bootstrap likelihood ratio test (BLRT). Lower AIC, BIC, and aBIC values, higher entropy values, and mean class assignment probabilities indicated a better fit [39]. A significant *p* value of LMR and BLRT indicated a better fit of the model compared with a model with one less class [40]. All indicators and interpretability were considered in determining the best fit model.

We used binary logistic regression to estimate the relationship between latent classes of sleep patterns and T2DM and calculated the odds ratios (ORs) and 95% confidence intervals (95% CIs). Potential confounding factors were selected based on the results of the univariate analyses. The missing data were processed by SPSS missing value analysis. All statistical tests were two-sided, and *p* values of <0.05 were considered statistically significant.

## 3. Results

### 3.1. Characteristics of Participants

A total of 1253 participants were recruited, among which 21 individuals failed to meet the inclusion criteria and 32 individuals failed to complete the questionnaire. A total of 1200 participants were included. The time required for participants to complete the face-to-face questionnaire survey ranged from 40 to 60 min. Of the 1200 participants included, aged 18 to 85 years, the mean (SD) age was 45.2 (14.8) years, 49.7% of the participants were female, and all participants were ethnic Han Chinese. There were 198 participants with T2DM, accounting for 16.5% of the total.

Compared with people without diabetes, those with diabetes were more likely to be older and to have a higher BMI and waist circumference, lower levels of education, lower annual household income, lower levels of physical activity, higher levels of stress and anxiety, and higher likelihood of restrained and emotional eating; more likely to suffer from hypertension, dyslipidemia, coronary heart disease, stroke, thyroid disease and osteoporosis; and more likely to be men, smokers, married and have a family history of diabetes. No significant differences were found between the two groups in the proportion of drinkers, external eating behaviors, levels of depression and neuroticism, or the prevalence of arthritis and cancer (Table 1).

### 3.2. Latent Classes of Sleep Patterns

According to the results of the model fit (Table 2), AIC, BIC, and aBIC gradually decreased as the number of classes increased, but the *p* value of LMR and BLRT was not significant in the 4-class model, and the entropy value was highest in the 3-class model. Considering these indicators holistically, we finally chose a 3-class model. The mean class assignment probability of each class was 0.89, 0.95, and 0.93 (for details, see Supplementary Table S1), indicating that individuals were classified into their most likely class with high certainty.

**Figure 1:**
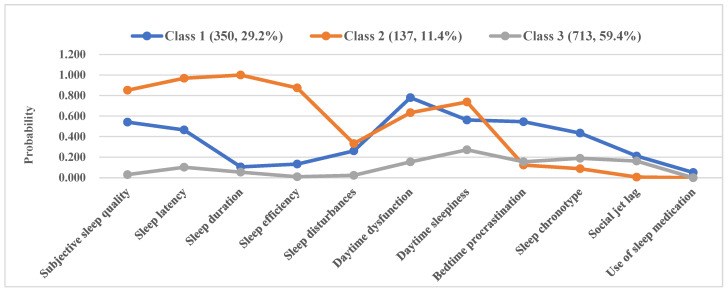
Graphical representation of the latent classes of sleep patterns.

As shown in Figure 1, class 1, class 2, and class 3 included 350 (29.2%), 137 (11.4%) and 713 (59.4%) participants, respectively. The abscissa is the 11 binary sleep indicators, and the ordinate is the probability that each class of people choose the worse one among the binary sleep indicators. Class 1 had the highest probability of severe bedtime procrastination, high SJL, night sleep chronotype, severe daytime dysfunction and use of sleep medicine, and moderate probability of poor subjective sleep quality, long sleep latency, severe sleep disturbances and daytime sleepiness; this class was designated as “circadian disruption with daytime dysfunction”. Class 2 had the highest probability of poor subjective sleep quality and sleep efficiency, long sleep latency, short sleep duration, severe sleep disturbances and daytime sleepiness, and moderate probability of daytime dysfunction, and was designated as “poor sleep status with daytime sleepiness”. Class 3 had a relatively favorable sleep status and was designated as “favorable sleep status”. There were significant differences in each dimension of sleep among the three sleep patterns, which showed great discriminative validity and further proved the accuracy of classification (Supplementary Table S2).

### 3.3. Differences of Participant Characteristics in 3 Classes of Sleep Patterns

Table 3 demonstrates the differences in sociodemographic, behavioral lifestyle, chronic diseases, and mental psychological state across participants with different sleep patterns. Among the 3 classes, class 1 had the lowest levels of physical activity as well as the highest proportion of unmarried individuals, education, restrained eating, emotional eating, external eating, depression and neuroticism; class 2 had the highest age, waist circumference, BMI, proportion of divorced or widowed individuals, prevalence of chronic diseases including dyslipidemia, coronary heart disease, stroke, arthritis and osteoporosis, and the lowest levels of education and annual household income; class 3 had the lowest stress, anxiety, and prevalence of hypertension and thyroid disease. Class 2 had a higher proportion of females and a higher prevalence of cancer than class 3.

### 3.4. Association between Latent Classes of Sleep Patterns and T2DM

Table 4 demonstrates the association between different sleep patterns and T2DM. Model 1 was a crude model which did not control for any variables, and its results showed a higher prevalence of T2DM in class 1 (OR: 1.68, 95% CI: 1.18–2.39) and class 2 (OR: 4.30, 95% CI: 2.84–6.52) compared to that of class 3. Model 2 controlled for age, sex, waist circumference, BMI, marital status, education, annual household income and family history of diabetes, and its results still showed that the prevalence of T2DM in class 1 (OR: 1.79, 95% CI: 1.19–2.69) and class 2 (OR: 2.81, 95% CI: 1.69–4.70) were significantly higher than that in class 3. Model 3 further controlled for smoking, physical activity levels, restrained eating and emotional eating; this model indicated that the results were still significant. The adjusted OR values of class 1 and class 2 were 2.00 (95% CI: 1.30–3.12) and 2.65 (95% CI: 1.57–4.47), respectively. Model 4 further controlled for chronic diseases that were significantly different in univariate analyses, namely, hypertension, dyslipidemia, coronary heart disease, stroke, thyroid disease, and osteoporosis, and found that the prevalence of T2DM in class 1 (OR: 1.80, 95% CI: 1.13–2.87) and class 2 (OR: 3.13, 95% CI: 1.78–5.51) were higher than that in class 3. Model 5 further controlled for stress and anxiety on the basis of the above adjusted factors. As a result, the prevalence of T2DM in class 2 (OR: 2.24, 95% CI: 1.26–4.00) was higher than that in class 3, while no significant differences were found between class 1 and class 3 (*p* = 0.541).

## 4. Discussion

Our study considered multiple aspects of sleep and constructed three latent classes of sleep patterns using LCA, namely, “circadian disruption with daytime dysfunction”, “poor sleep status with daytime sleepiness”, and “favorable sleep status”. In addition, we found that “poor sleep status with daytime sleepiness” was significantly associated with T2DM.

We found that there were aggregated characteristics of sleep problems. For example, poor sleep quality, low sleep efficiency, insufficient sleep duration, difficulty falling asleep and daytime sleepiness often coexisted, and this was highly similar to the findings of Chen et al. [41]. However, the previous study did not analyze daytime sleepiness. The aggregated characteristics of these sleep problems are traceable, as difficulty falling asleep may reduce sleep duration and sleep efficiency, leading to various sleep problems, including daytime sleepiness. By the same token, relief of difficulty falling asleep, sleep disturbance, adequate sleep duration and high sleep efficiency improves subjective sleep quality, use of sleep medication, daytime sleepiness and function, and maintains a relatively good sleep status, similar to the population with “favorable sleep status” in our study. Most previous research analyzed sleep patterns based only on indicators related to sleep quality, such as sleep duration, sleep efficiency, insomnia and daytime status [41,42]. We also considered sleep behavior habits, such as bedtime procrastination, SJL, and sleep chronotype, and found that bedtime procrastination, night sleep chronotype, higher SJL and daytime dysfunction often coincide. This may be attributed to the fact that bedtime procrastination may lead to a night sleep chronotype, in which individuals tend to accumulate more sleep debt and create higher SJL, which causes circadian disruption. Circadian disruption can lead to symptoms of daytime fatigue and poor mental agility, which can further exacerbate daytime dysfunction [43].

The sleep pattern of “poor sleep status with daytime sleepiness” is predominantly distributed in older adults, and the involved sleep problems are highly consistent with a series of physiological sleep changes in the normal aging process [44]. In addition, the sleep pattern is more common in elderly women, which may be related to the decreased levels of progesterone and estrogen after menopause which normally facilitate normal sleep patterns [45]. The sleep pattern is also more prevalent in patients with multiple chronic diseases. This may be due to the fact that the populations in the sleep pattern were of older age and had multiple sleep disorders, which have been shown to be risk factors for chronic diseases, and in turn chronic diseases may also contribute to poor sleep status [46,47,48]. Consistent with previous studies, the population with various sleep disorders had higher waist circumference and BMI, associated with overweight obesity, which may be related to increased food intake due to decreased concentrations of satiety-promoting gut hormone glucagon-like peptide 1 (GLP-1) caused by sleep disorders [49,50]. This sleep pattern was associated with T2DM in our study, which may be related to multiple mechanisms, such as hormones (changes in appetite-regulating hormones, melatonin), the nervous system (activity of the sympathetic nervous system and hypothalamic-pituitary-adrenergic axis), and inflammatory factors [51,52,53]. The long-term cumulative negative effects of these sleep problems may be associated with T2DM. Therefore, it is necessary to screen for populations with the sleep pattern early, especially in older adults, women, and those with higher waist circumference and BMI and a greater prevalence of chronic diseases. Timely interventions for various sleep problems may reduce adverse health outcomes.

The sleep pattern of “circadian disruption with daytime dysfunction” was mainly found in the younger population. Young adults tend to delay bedtime due to work stress and excessive use of smartphones, causing circadian disruption and daytime dysfunction [54], followed by longer rest-day sleep duration to compensate for lack of sleep on weekdays, resulting in higher SJL [43]. In addition, this sleep pattern has higher levels of anxiety and stress. Stress and anxiety have been confirmed to be related to T2DM [55,56], and this relationship was also found in our study. This may explain why the association between the sleep pattern of “circadian disruption with daytime dysfunction” and T2DM became nonsignificant after adjusting for stress and anxiety. In previous studies, the sleep behavior habits associated with circadian disruption, such as bedtime procrastination, night sleep chronotype, and SJL in this pattern, were shown to be independently associated with T2DM. They may play an important role in glucose regulation through melatonin secretion and the peripheral clock that generates tissue-specific rhythmic gene expression [57,58]. However, the relationship between a certain sleep dimension and T2DM was also influenced by other sleep dimensions within the same sleep pattern. In our study, although the sleep pattern of “circadian disruption with daytime dysfunction” involved issues independently associated with T2DM including circadian disruption, daytime dysfunction, and moderate sleep quality, having adequate sleep duration and higher sleep efficiency may change the association between these sleep problems and T2DM [59]. This may be a compensatory self-protection mechanism of the body. Further research should investigate whether and what changes will occur in this compensatory outcome if the individual is exposed to various sleep problems for a long period. Therefore, it is necessary to prospectively trace the relationship between sleep patterns and T2DM and elucidate the underlying mechanism.

In this study, we used LCA to analyze the complex associations between sleep dimensions, and we categorized participants into three classes of sleep patterns according to these associations. In addition, we considered confounding factors, such as sociodemographic, behavioral lifestyle, chronic diseases and mental psychological state, and found that “poor sleep status with daytime sleepiness” was significantly associated with T2DM. This explained the real-world relationship between sleep and T2DM accurately and comprehensively and may serve as a practical guide. Nevertheless, there were several limitations in our study. First, the study was unable to determine a causal relationship between sleep patterns and T2DM due to its cross-sectional design; however, the significant correlation found in our study provides a basis for future prospective analyses of this relationship. Second, since this was a large sample survey, all sleep dimensions were measured by subjective self-reports. Hence, although the measurement tools had good reliability and validity, bias may exist. Finally, because the survey was conducted in Xuzhou city, Jiangsu Province, China, and all participants were ethnic Han Chinese, extrapolation of the findings to other regions and ethnic groups is potentially limited.

In the future, objective sleep measures can be used to investigate latent classes of sleep patterns in multicenter populations based on LCA, and the causal relationship between each sleep pattern and T2DM can be prospectively analyzed. Future research may be used to inform intervention programs and validate the effects of unhealthy sleep patterns on T2DM. 

The findings of our study suggest that health care institutions, especially those involved in primary care, should pay attention to the impact of sleep on health and routinely assess the characteristics of sleep patterns of residents. Particular attention should be given to the population exhibiting “poor sleep status with daytime sleepiness”, and early intervention strategies should be formulated. Healthy sleep patterns should be promoted and publicized to improve the overall sleep health of all populations.

## 5. Conclusions

Aggregated features exist between multiple sleep problems. Sleep patterns with sleep problems such as poor subjective sleep quality and sleep efficiency, long sleep latency, short sleep duration, severe sleep disturbance and daytime sleepiness, and moderate daytime dysfunction were significantly associated with T2DM. The causal relationship should be further verified by longitudinal studies in the future.

## Figures and Tables

**Table 1:** Characteristics of participants.

Characteristic	Total	No-DM	DM	*p*-Value
Age (years)	45.2 ± 14.8	43.21 ± 14.04	55.22 ± 14.21	<0.001 **
WC (cm)	79.5 ± 12.2	78.38 ± 12.07	84.90 ± 11.25	<0.001 **
BMI (kg/m^2^)	23.9 ± 3.7	23.69 ± 3.61	24.92 ± 3.67	<0.001 **
Sex				<0.001 **
Male	604 (50.3)	478 (47.7)	126 (63.6)	
Female	596 (49.7)	524 (52.3)	72 (36.4)	
Marital status				<0.001 **
Unmarried	162 (13.5)	156 (15.6)	6 (3.0)	
Married	927 (77.3)	750 (74.9)	177 (89.4)	
Divorced or widowed	111 (9.3)	96 (9.6)	15 (7.6)	
Education				<0.001 **
Junior high school and below	570 (47.5)	452 (45.1)	118 (59.6)	
High school and above	630 (52.5)	550 (54.9)	80 (40.4)	
Annual household income (RMB)				<0.001 **
<80,000 RMB	704 (58.7)	556 (55.5)	148 (74.7)	
80,000- RMB	426 (35.5)	384 (38.3)	42 (21.2)	
>300,000 RMB	70 (5.8)	62 (6.2)	8 (4.0)	
Smoking (yes)	249 (20.8)	193 (19.3)	56 (28.3)	0.004 **
Alcohol (yes)	268 (22.3)	214 (21.4)	54 (27.3)	0.068
Physical activity level				<0.001 **
Low	584 (50.3)	502 (52.0)	82 (41.8)	
Moderate	393 (33.9)	299 (31.0)	94 (48.0)	
High	184 (15.8)	164 (17.0)	20 (10.2)	
Sleep duration	7.19 ± 1.58	7.4 ± 1.5	6.3 ± 1.5	<0.001 **
PSQI ^#^	5.77 ± 3.84	5.3 ± 3.6	8.3 ± 4.1	<0.001 **
ESS	8.68 ± 5.36	8.0 ± 5.1	12.1 ± 5.4	<0.001 **
BPS ^#^	24.59 ± 6.29	24.9 ± 5.9	23.1 ± 7.8	<0.001 **
SJL ^#^	0.35 ± 0.59	0.4 ± 0.6	0.18 ± 0.5	<0.001 **
Sleep chronotype				<0.001 **
Morning	240 (20.0)	181 (18.1)	59 (29.8)	
Intermediate	670 (55.8)	560 (55.9)	110 (55.6)	
Night	290 (24.2)	261 (26.0)	29 (14.6)	
DEBQ	68.9 ± 21.4	70.09 ± 21.93	62.76 ± 17.54	<0.001 **
Restrained eating ^#^	22.1 ± 9.0	22.60 ± 9.06	19.67 ± 7.98	<0.001 **
Emotional eating ^#^	21.5 ± 10.2	22.09 ± 10.46	18.67 ± 8.11	<0.001 **
External eating	25.2 ± 8.6	25.39 ± 8.56	24.42 ± 8.47	0.144
DASS-21 ^#^	10.5 ± 9.3	9.70 ± 8.90	14.55 ± 10.28	<0.001 **
Stress ^#^	5.0 ± 4.0	4.49 ± 3.66	7.58 ± 4.65	<0.001 **
Anxiety ^#^	2.9 ± 3.1	2.63 ± 3.00	4.32 ± 3.44	<0.001 **
Depression ^#^	2.6 ± 3.3	2.58 ± 3.26	2.66 ± 3.54	0.547
Neuroticism	20.6 ± 8.9	20.65 ± 8.86	20.46 ± 9.15	0.782
Hypertension	222 (18.5)	150 (15.0)	72 (36.4)	<0.001 **
Dyslipidemia	176 (14.7)	103 (10.3)	73 (36.9)	<0.001 **
CHD	28 (2.3)	12 (1.2)	16 (8.1)	<0.001 **
Stroke	65 (5.4)	20 (2.0)	45 (22.7)	<0.001 **
Arthritis	101 (8.4)	79 (7.9)	22 (11.1)	0.135
Osteoporosis	112 (9.3)	85 (8.5)	27 (13.6)	0.023 *
Cancer	4 (0.3)	3 (0.3)	1 (0.5)	0.646
Thyroid disease	92 (7.7)	57 (5.7)	35 (17.7)	<0.001 **
Family history of diabetes	229 (19.1)	140 (14.0)	89 (44.9)	<0.001 **

Abbreviations: BMI: body mass index, BPS: Bedtime Procrastination Scale, CHD: coronary heart disease, DASS: Depression, Anxiety and Stress Scales, DEBQ: Dutch Eating Behavior Questionnaire, DM: diabetes mellitus, ESS: Epworth Sleepiness Scale, PSQI: Pittsburgh Sleep Quality Index, SJL: social jet lag, WC: waist circumference. Notes: ^#^ Mann–Whitney U test, * *p* < 0.05, ** *p* < 0.01. Continuous variables: Student’s *t*-test; Categorical variables: Pearson’s χ^2^-tests.

**Table 2:** Fit statistics for latent class models.

Number of Classes	K	Loglikelihood	AIC	BIC	aBIC	Entropy	LMR	BLRT
1	11	−6596.259	13,214.517	13,270.508	13,235.568			
2	23	−5991.212	12,028.423	12,145.495	12,072.438	0.782	<0.001	<0.001
3	35	−5777.129	11,624.257	11,802.410	11,691.236	0.811	<0.001	<0.001
4	47	−5631.602	11,357.204	11,596.437	11,447.147	0.804	0.1306	0.1329

Abbreviations: AIC: Akaike information criterion, BIC: Bayesian information criterion, aBIC: sample size-adjusted Bayesian information criterion, LMR: Vuong-Lo-Mendell-Rubin, BLRT: bootstrap likelihood ratio test.

**Table 3:** Participants characteristics in 3 classes of sleep patterns.

Characteristics	Class 1 (*n* = 350)	Class 2 (*n* = 137)	Class 3 (*n* = 713)	*p*-Value	Post Hoc
Age (years)	41.91 ± 15.80	60.38 ± 12.23	43.88 ± 12.81	<0.001 **	2 > 3 > 1
WC (cm)	78.99 ± 13.27	82.07 ± 10.74	79.18 ± 11.83	0.015 *	2 > 1, 3
BMI (kg/m^2^)	23.72 ± 3.96	25.61 ± 3.66	23.65 ± 3.39	<0.001 **	2 > 1, 3
Sex				0.014 *	
Male	167 (47.7)	56 (40.9)	381 (53.4)		2 < 3
Female	183 (52.3)	81 (59.1)	332 (46.6)		2 > 3
Marital status				<0.001 **	
Unmarried	76 (21.7)	1 (0.7)	85 (11.9)		1 >3 > 2
Married	256 (73.1)	107 (78.1)	564 (79.1)		
Divorced or widowed	18 (5.1)	29 (21.2)	64 (9.0)		2 > 1, 3
Education				<0.001 **	
Junior high school and below	109 (31.1)	107 (78.1)	354 (49.6)		2 > 3 > 1
High school and above	241 (68.9)	30 (21.9)	359 (50.4)		1 > 3 > 2
Annual household income (RMB)				<0.001 **	
<80, 000 RMB	180 (51.4)	115 (83.9)	409 (57.4)		2 > 1, 3
80, 000- RMB	153 (43.7)	19 (13.9)	254 (35.6)		1 > 3 > 2
>300, 000 RMB	17 (4.9)	3 (2.2)	50 (7.0)		
Smoking (yes)	77 (22.0)	24 (17.5)	148 (20.8)	0.548	
Alcohol (yes)	78 (22.3)	24 (17.5)	166 (23.3)	0.333	
Physical activity level				<0.001 **	
Low	201 (58.6)	53 (38.7)	330 (48.5)		1 > 2, 3
Moderate	78 (22.7)	63 (46.0)	252 (37.0)		1 < 2, 3
High	64 (18.7)	21 (15.3)	99 (14.5)		
DEBQ	76.85 ± 22.81	63.62 ± 17.07	65.97 ± 20.45	<0.001 **	1 > 2, 3
Restrained eating	25.37 ± 9.66	19.47 ± 6.16	21.03 ± 8.62	<0.001 **	1 > 2, 3
Emotional eating	24.36 ± 12.06	20.07 ± 7.75	20.42 ± 9.30	<0.001 **	1 > 2, 3
External eating ^‡^	27.11 ± 8.53	24.08 ± 7.81	24.53 ± 8.56	<0.001 **	1 > 2, 3
DASS-21	14.46 ± 9.51	13.26 ± 8.82	8.03 ± 8.46	<0.001 **	3 < 1, 2
Stress	6.37 ± 4.07	7.25 ± 4.00	3.89 ± 3.56	<0.001 **	3 < 1, 2
Anxiety	4.29 ± 3.25	3.72 ± 3.30	2.08 ± 2.75	<0.001 **	3 < 1, 2
Depression	3.80 ± 3.48	2.29 ± 2.83	2.06 ± 3.16	<0.001 **	1 > 2, 3
Neuroticism ^‡^	24.94 ± 8.32	22.47 ± 8.74	18.14 ± 8.32	<0.001 **	1 > 2 > 3
Hypertension	80 (22.9)	45 (32.8)	97 (13.6)	<0.001 **	3 < 1, 2
Dyslipidemia	45 (12.9)	42 (30.7)	89 (12.5)	<0.001 **	2 > 1, 3
CHD	9 (2.6)	11 (8.0)	8 (1.1)	<0.001 **	2 > 1, 3
Stroke	17 (4.9)	19 (13.9)	29 (4.1)	<0.001 **	2 > 1, 3
Arthritis	33 (9.4)	41 (29.9)	27 (3.8)	<0.001 **	2 > 1> 3
Osteoporosis	29 (8.3)	43 (31.4)	40 (5.6)	<0.001 **	2 > 1, 3
Cancer	2 (0.6)	2 (1.5)	0 (0.0)	0.016 *	2 > 3
Thyroid disease	47 (13.4)	13 (9.5)	32 (4.5)	<0.001 **	3 < 1, 2

Abbreviations: BMI: body mass index, CHD: coronary heart disease, DASS: Depression, Anxiety and Stress Scales, DEBQ: Dutch Eating Behavior Questionnaire, DM: diabetes mellitus, WC: waist circumference. Notes: ^‡^ One-way Anova, * *p* < 0.05, ** *p* < 0.01. Continuous variables: Kruskal–Wallis test; Categorical variables: Pearson’s χ^2^-tests.

**Table 4:** Logistic analyses of the association between latent classes of sleep patterns with T2DM.

Class	Model 1		Model 2		Model 3		Model 4		Model 5	
	OR (95% CI)	*p*	OR (95% CI)	*p*	OR (95% CI)	*p*	OR (95% CI)	*p*	OR (95% CI)	*p*
Class 3	1.00		1.00		1.00		1.00		1.00	
Class 1	1.68 (1.18–2.39)	0.004	1.79 (1.19–2.69)	0.005	2.00 (1.30–3.12)	0.002	1.80 (1.13–2.87)	0.013	1.17 (0.70–1.85)	0.541
Class 2	4.30 (2.84–6.52)	<0.001	2.81 (1.69–4.70)	<0.001	2.65 (1.57–4.47)	<0.001	3.13 (1.78–5.51)	<0.001	2.24 (1.26–4.00)	0.006

Abbreviations: OR: odds ratio, CI: confidence interval. Model 1: no adjusted; model 2: adjusted for age, sex, BMI, WC, marital status, education, annual household income and family history of diabetes; model 3: on the basis of model 2, smoking, physical activity level, restrained and emotional eating were further adjusted; model 4: on the basis of model 3, hypertension, dyslipidemia, coronary heart disease, stroke, osteoporosis, and thyroid disease were further adjusted; model 5: on the basis of model 4, stress and anxiety were further adjusted.

## Data Availability

The data presented in this study are available on request from the corresponding author.

## Supplementary Materials

The following supporting information can be downloaded at: https://www.mdpi.com/article/10.3390/ijerph20010393/s1, Supplementary Table S1: Mean class assignment probabilities of 3 classes; Supplementary Table S2: Sleep characteristics in 3 classes of sleep patterns.
