# Supplementary material for: Association of Sleep Patterns with Type 2 Diabetes Mellitus: A Cross-Sectional Study Based on Latent Class Analysis
Source: Int J Environ Res Public Health. 2022 Dec 26;20(1):393. doi: 10.3390/ijerph20010393 (PMC9819015; doi:10.3390/ijerph20010393)
Supplement: Supplementary file 1 [file ijerph-20-00393-s001.zip › ijerph-2073770-supplementary.pdf]

Supplementary Table S1. Mean class assignment probabilities of 3 classes

Latent class	Class 1 (%)	Class 2 (%)	Class 3 (%)
Class 1	0.891	0.008	0.101
Class 2	0.047	0.951	0.002
Class 3	0.072	0.001	0.927

Supplementary Table S2. Sleep characteristics in 3 classes of sleep patterns.

Characteristic	Class 1 (*n* = 350)	Class 2 (*n* = 137)	Class 3 (*n* = 713)	*P*-value
PSQI	7.77±2.47	12.85±2.13	3.42±1.95	<0.001**
Subjective sleep quality				<0.001**
0	19 (5.4)	2 (1.5)	223 (31.3)	
1	126 (36.0)	17 (12.4)	478 (67.0)	
2	175 (50.0)	89 (65.0)	12 (1.7)	
3	30 (8.6)	29 (21.2)	0 (0.0)	
Sleep latency (min)				
Workday	29.65±26.47	100.47±50.90	19.67±15.27	<0.001**
Free day	30.19±29.85	100.84±50.68	19.92±15.43	<0.001**
Sleep duration (h)	7.19±1.27	4.61±0.77	7.68±1.32	<0.001**
Sleep efficiency (%)	85.13±9.55	66.16±9.35	90.67±6.45	<0.001**
ESS	10.59±5.40	12.50±4.67	7.00±4.75	<0.001**
SJL	0.47±0.73	0.04±0.14	0.35±0.54	<0.001**
BPS	28.58±6.51	23.58±5.63	22.82±5.35	<0.001**
Sleep chronotype				<0.001**
Morning	30 (8.6)	36 (26.3)	174 (24.4)	
Intermediate	168 (48.0)	88 (64.2)	414 (58.1)	
Night	152 (43.4)	13 (9.5)	125 (17.5)	
Sleep disturbances				<0.001**
0	12 (3.4)	1 (0.7)	179 (25.1)	
1	240 (68.6)	90 (65.7)	521 (73.1)	
2	77 (22.0)	42 (30.7)	13 (1.8)	
3	21 (6.0)	4 (2.9)	0 (0.0)	
Daytime dysfunction				<0.001**
0	23 (6.6)	9 (6.6)	357 (50.1)	
1	44 (12.6)	40 (29.2)	247 (34.6)	
2	160 (45.7)	36 (26.3)	75 (10.5)	
3	123 (35.1)	52 (38.0)	34 (4.8)	
Use of sleep medication (yes)	30 (8.6)	11 (8.0)	9 (1.3)	<0.001**

Abbreviations: BPS Bedtime Procrastination Scale, ESS Epworth Sleepiness Scale, PSQI Pittsburgh Sleep Quality Index, SJL social jet lag. Notes: * $p < 0.05$, ** $p < 0.01$. Continuous variables: Kruskal-Wallis test; Categorical variables: Pearson's $\chi 2$-tests.
